# Changes in hoof morphology in racing Thoroughbreds during a period of ninety days unshod

**DOI:** 10.3389/fvets.2026.1863433

**Published:** 2026-10-01

**Authors:** Gavin R. Moody, Simon J. Curtis, A. J. Northrop, Jaime H. Martin

**Affiliations:** 1 Gavin Moody Farriers Ltd, Red lodge, Bury St Edmunds, United Kingdom; 2 Independent researcher, Bury St Edmunds, United Kingdom; 3 School of Animal, Rural and Environmental Sciences, Nottingham Trent University, Southwell, United Kingdom

**Keywords:** hoof morphology, racehorses, regular trimming, restoration, unshod

## Abstract

**Background:**

Low hoof angles and discrepancies in the dorsal heel are linked to musculoskeletal breakdown, suspensory apparatus failure, and retirement in Thoroughbred racehorses. Evidence-based research on correcting hoof adaptations is limited, which leads to a reliance on therapeutic shoeing.

**Objectives:**

This study aimed to test the hypothesis that morphological changes occur during active unshod training and that trimming immediately affects hoof angles.

**Study design:**

This is a controlled, quantitative, longitudinal study that uses a convenience sample.

**Methods:**

A total of 36 racehorses were divided into an unshod treatment group (*n* = 23) and a control group (*n* = 13) that remained shod between hoof-care cycles. Trimming followed a recognised standard. Photogrammetry was used to compare initial, endpoint, pre-, and post-trim lateral and solear images.

**Results:**

Trimming improved the dorsal hoof wall angle (DHWA), heel angle (HA), and the DHWA-HA difference (*p* = 0.001) and altered solear morphometrics (*p* = 0.001, treatment; *p* = 0.003, control). Quantified longitudinal, pre-trim treatment data at the end of the 90-day study revealed that the DHWA remained stable (51.22 ± 3.28° to 51.31 ± 3.03°), while the HA increased (29.97 ± 8.81° to 34.62 ± 7.77°) (*p* = 0.005), and the DHWA-HA difference decreased (21.25 ± 6.90° to 16.69 ± 6.44°) (*p* = 0.001). Control group results were non-significant. A moderate negative correlation between the DHWA and age (*r* = −0.412, *p* = 0.001) indicated that the DHWA decreased as the horses aged.

**Main limitations:**

The study utilised a convenience cohort of racehorses; a larger control group was preferred.

**Conclusion:**

A 90-day period of unshod training restores hoof morphology, offering non-invasive solutions to mitigate hoof-angle-related injury, early retirement, and financial loss. Increased HA and a decreased DHWA-HA difference suggests that the stimulation of the frog, digital cushion (DC), and neuroreceptors drives hoof remodelling. Farriers are often pressured to manipulate the DHWA through trimming the dorsum, this study provides evidence that heel angle, and palmar hoof function should be prioritised. Immediate post-trim improvements imply that greater deterioration occurred during the hoof-care cycle in the control group, indicating that shorter cycles may be necessary for optimal hoof morphology. The findings show that age-related reduction in the DHWA persists for longer than has previously been reported.

## Introduction

Hoof morphology and balance directly influence locomotion and soundness in athletic horses, with the equine foot being a common site of lameness (1, 2). In Thoroughbred racehorses, poor hoof conformation, specifically long toes and under-run heels, has been associated with injury and musculoskeletal breakdown (3). Additionally, suspensory apparatus failure (SAF) has been associated with disparities between the dorsal hoof wall angle (DHWA) and heel angle (HA) (1, 3). Furthermore, steel shoes have been shown to prolong break-over duration compared to unshod hooves, increasing the mechanical strain on the distal limb (4). Although hoof capsule distortion is frequently linked to injury and lameness, the relationship is not always straightforward (5, 6). The most common cause of early retirement amongst racehorses is lameness (7). Beyond the implications for welfare and longevity, the economic impact of premature retirement necessitates the write-off of substantial training investment, potential career earnings, and future breeding value. Periods of barefoot management have been proposed to mitigate hoof distortion and restore structural integrity (8); however, little research has been conducted to investigate the effects of shoe removal on hoof morphology in actively training Thoroughbred racehorses.

Hoof morphology adapts in response to loading conditions over time. Conformation, ground reaction forces, shoeing, and speed training all influence the loads experienced by the hoof and may contribute to hoof capsule distortion and its associated pathology (6). This is of relevance to Thoroughbred racehorses, which enter training at a young age and commonly experience high ground reaction forces and are shod throughout their racing careers. Previous work has shown that hoof angles may deteriorate during periods of intensive training in Thoroughbred racehorses (9). Consequently, further research is required to identify factors that influence hoof morphology and to inform evidence-based preventative strategies aimed at mitigating reductions in hoof angles within this population.

Hoof conformation adapts throughout their growth and development as hoof structure and composition change over time (10). Research involving Thoroughbred foals has demonstrated the effects of loading on hoof wall growth and shape; weanlings possess a relatively steep DHWA, which decreases with maturation (5). The DHWA has been reported to decline from 6 months to 2 years of age (11); however, it remains unclear whether this trend continues beyond 2 years.

Thoroughbred yearlings are commonly shod before entering training to help manage the mechanical demands of race training. Proske et al. (12) suggested that shoeing reduces the interaction between the frog, sole, and the ground, potentially influencing the development of the digital cushion. These findings raise the possibility that prolonged shoeing may affect hoof development and morphology from an early age by prioritising early training over physical development. However, evidence on actively training Thoroughbred racehorses remains limited.

Hoof morphology has been described as changing over the course of a shoeing cycle in working horses, during which toe growth increased solear weight-bearing length and reduced the heel angle (HA), resulting in a more negative hoof pastern axis (13). Morphological changes within just one shoeing cycle support previous suggestions that regular hoof care as frequently as every 3 weeks may be required to minimise hoof distortion and maintain balance in racehorses (6).

Although farriery can affect the hoof shape over a short term, conformation and loading patterns are also likely to substantially contribute to hoof morphology (5).

During objective and subjective assessments of Thoroughbreds, heel distortion, as a measure of hoof shape, is frequently evaluated by comparing the DHWA and HA. Published guidelines vary regarding ideal alignment, with Colles (14) suggesting that these angles should differ by no more than 1° or 2°, whereas Turner et al. (2) accept a variance of up to approximately 5°. However, heel angles >5° below the DHWA are often referred to as underrun heels (1). Low heel angles and collapsed heel conformation are commonly reported in racehorses and have been associated with reduced hoof balance and increased injury risk, emphasising the need to better understand methods of restoring heel morphology.

The immediate changes in hoof morphology following trimming have been assessed using radiographic and photographic techniques. Radiographic assessments of Warmblood horses following trimming demonstrated modest reductions in hoof length (15). Similarly, Antonioli et al. (16) reported immediate changes in the DHWA and HA; however, the interpretation of these findings is complicated by a simultaneous reduction in the heel angle in the same horses. Recorded morphological shifts from pre- to post-trim may highlight the necessity of standardised, non-invasive photography to monitor baseline angles before dynamic loading.

In addition to immediate post-trim shifts, hoof morphology adapts to load, exercise intensity, shoeing state, and ground reaction forces. In a seven-week study of shod and barefoot Quarter horses, Malone and Davies (3) reported an increase in DHWA in barefoot horses compared with a decrease in shod controls. Similarly, a study involving seven Arabian horses reported positive results after lowering the heels to engage the frog and bars during weight bearing whilst maintaining the mediolateral balance.

Biomechanical changes within the foot were suggested to respond to locomotor forces, resulting in palmar heel expansion and increased HA and support length (17). Peel et al. (9) observed short-term changes in the hoof angle according to training status, demonstrating a more favourable angle once gallop training ceased. These findings indicate that hoof morphology responds to mechanical loading and workload variation over short periods, contrasting with theories that hoof distortion occurs over prolonged periods whilst in a semi-static state (6). In view of these findings, it may be helpful for hoof-care strategies to account for individual training schedule demands.

The presence of aggrecanase-1 in the lamellae provides indirect evidence of the importance of frog interaction with the substrate during weight bearing. These extracellular molecules are associated with tissue that requires compressive loading, suggesting that it is beneficial for lamellae to be loaded through engagement with the substrate (17).

The digital cushion (DC) plays a central role in load dissipation within the hoof capsule. In young horses, the structural composition of the DC consists predominantly of adipose tissue, which progressively remodels into fibrocartilage with maturity, an adaptation driven by mechanical loading and ground interactions (10). Studies of barefoot horses have suggested that increased frog and sole contact with the ground promotes normal hoof function and development as specialised mechanoreceptors packed within and around the DC respond to vibrations from the ground and subsequently influence vascular activity (18).

Supporting this, Proske et al. (12) demonstrated adaptations in DC depth over a 140-day barefoot period using ultrasonography. Furthermore, the viscoelastic properties of hoof horn result in gradual deformation under sustained loading (“creep”), which may contribute to changes in the hoof shape over time (5). Collectively, these findings suggest that shoeing and hoof–ground interaction may influence hoof morphology, providing a potential mechanism for the changes observed in barefoot horses. Consequently, traditional practices may require revision to incorporate evidence-based hoof-care strategies that optimise hoof-substrate interaction and reduce long-term structural compromises.

The aim of this study was to investigate the effect of shoe removal on hoof morphology and hoof angle in a cohort of racehorses in active training over a period of 90 days. The objective was to measure and compare the hooves of racehorses using photographic images. The images were independently measured, comparing the treatment group (unshod) with the control group (shod).

It was hypothesised that (1) morphological and hoof-angle changes can be restored during a period of 90 days unshod and that (2) trimming would result in immediate changes to hoof angles.

## Materials and methods

A quantitative method of longitudinal research was undertaken involving racehorses (*n* = 36) selected by convenience sampling following the trainer’s pre-established training directives, independent of the study protocol. All horses were healthy and scheduled to remain in active training; 23 of them were designated as non-racing horses during the anticipated study period by the trainer, leaving 13 others that could possibly race. This resulted in two discrete groups for the study; however, no horses raced over the course of the study. Both groups were trained utilising turf and synthetic surfaces, horse walkers, and treadmills. No horses were turned out in the paddock.

The treatment group (*n* = 23) had their shoes removed at the beginning of the study and were trimmed by one author to a recognised standard over the course of 90 days. Digital photography was used at each farriery cycle. The control group (*n* = 13) underwent the same trimming methodology but remained shod with fullered concave steel shoes (19 mm × 8 mm).

All horses were in good health at the beginning of the study, with no signs of conditions such as hoof cracks, flares, or balance distortion. The ages of the selected horses ranged from 2 to 4 years at the start of the study to 3–5 years at the end. This was because the study spanned the years 2023 and 2024, with Thoroughbreds considered to age up from 1 January.

The horses were positioned on a level, clean surface and held by an assistant while their hooves were cleaned. A podoblock was not utilised due to occupational health and safety considerations related to handling of young horses within a professional training environment. A 12.7-mm circular calibration sticker was placed on the lateral hoof wall of both fore hooves, at the midsection of the widest point of the hoof, before trimming, and the horses were photographed while standing evenly. The camera was positioned at a focal distance of 304 mm perpendicular to the lateral HPA and the hoof capsule. The camera lens was maintained at a height of 46 mm above the firm substrate, aligned horizontally with the centre of the lateral hoof wall, with the heel bulbs aligned and the calibration sticker centred on the screen (Figure 1).

**Figure 1:**
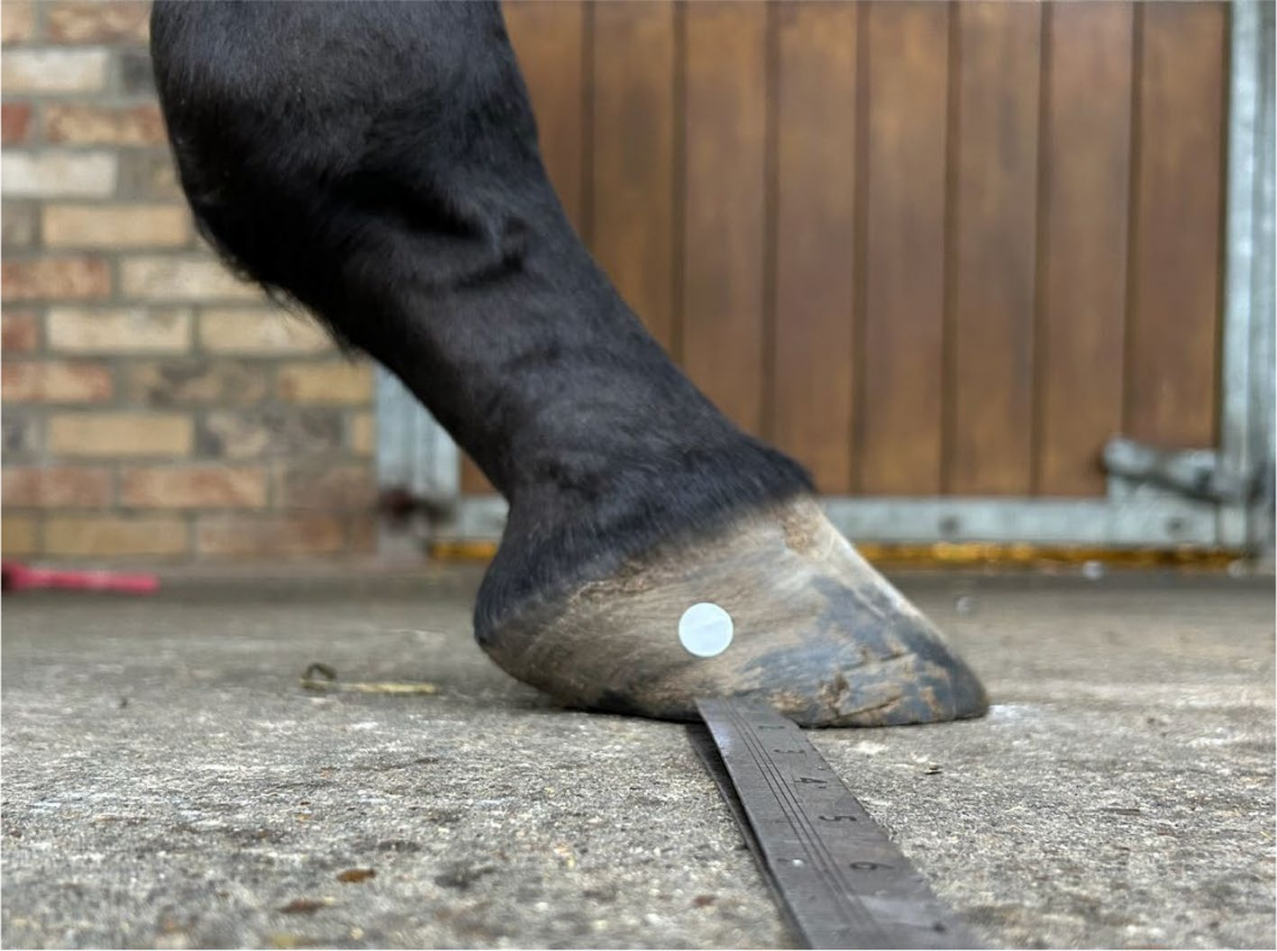
The lateral position of the camera, perpendicular to the HPA, with the heel bulbs aligned and a calibration sticker affixed at the widest point of the hoof.

A 12.7 mm calibration sticker was then affixed to the solear surface of each hoof pre-trim, directly in front of the apex of the frog. Each forelimb was held steady by an assistant, with the solear surface of the hoof positioned parallel to the camera at a distance of approximately 300 mm. Five sequential photographs encompassing the whole ground-bearing surface were taken per hoof, allowing for subsequent selection of an optimal image free from perspective distortion (Figure 2).

**Figure 2:**
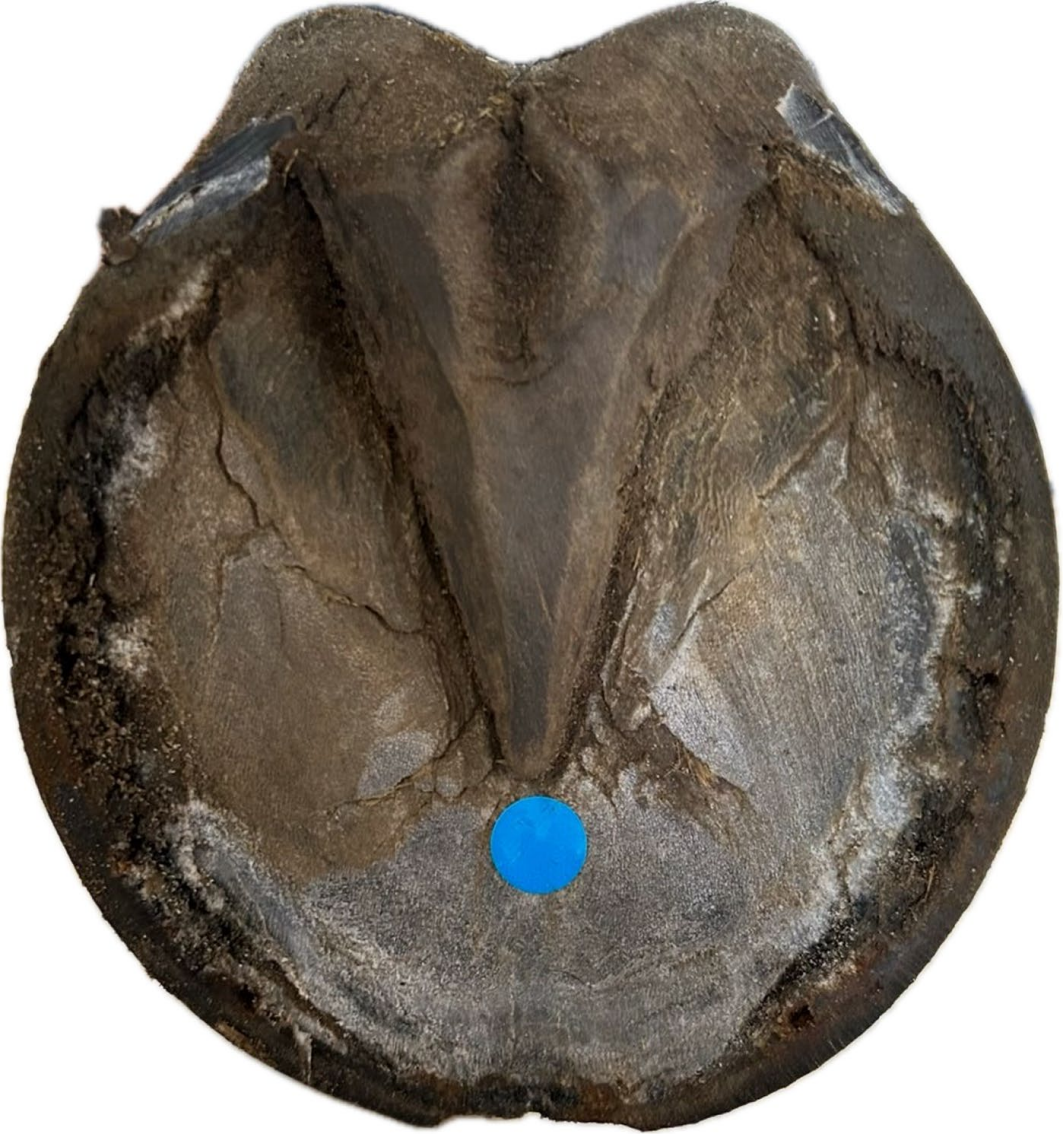
Photograph of the solear view, with the calibration sticker affixed directly in front of the apex of the frog.

The hoof was trimmed using a repeatable trimming protocol as follows:

1. The frogs were trimmed symmetrically, enabling heel height on both the medial and lateral sides to be assessed, along with solear hoof shape.
2. Any exfoliating sole was removed, and the bars were shaped.
3. The hoof wall was trimmed to a flat, level plane perpendicular to the long axis of the cannon.
4. A slight bevel was applied to the hoof wall of the treatment group to prevent hoof breaks.
5. The same process of photography was repeated post-trim and throughout the 90-day study at each farriery cycle, for both the treatment and control groups.

The trimming and shoeing of all horses in the study were performed by one author, as close to a 30-day cycle as possible. Photographic images of each horse were compiled into individual albums and saved with the date and type of view of each farriery cycle. Images were originally captured using an iPhone 14 and then transferred and uploaded to Metron-Hoof (MH).

An independent objective evaluation of each image was undertaken by another author, who was blinded to group assignment. Using MH software, repeatable points were placed on the images. Markers were placed on the lateral images at the proximal and distal borders of the DHWA and HA, and a surface line was marked indicating the weight-bearing surface parallel to the ground, enabling the measurement of the DHWA and lateral heel angle of both pre-trim and post-trim hooves (Figure 3).

**Figure 3:**
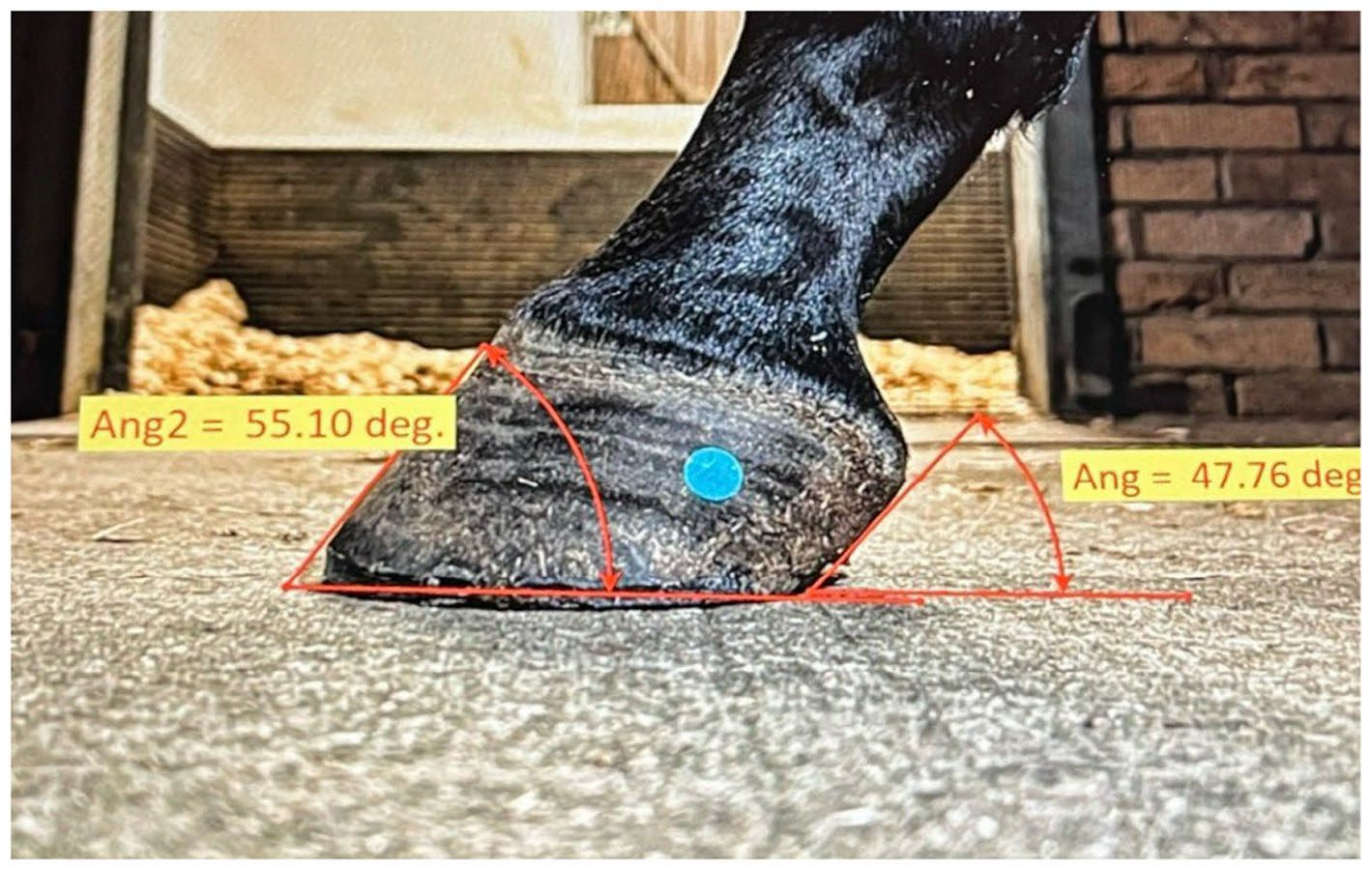
The DHWA and HA were identified using image measuring software. The horizontal line represented the same weight-bearing surface for both angles.

Markers were placed at clearly defined repeatable points on the sole to calibrate solear morphometric measurements in centimetres (Figure 4).

**Figure 4:**
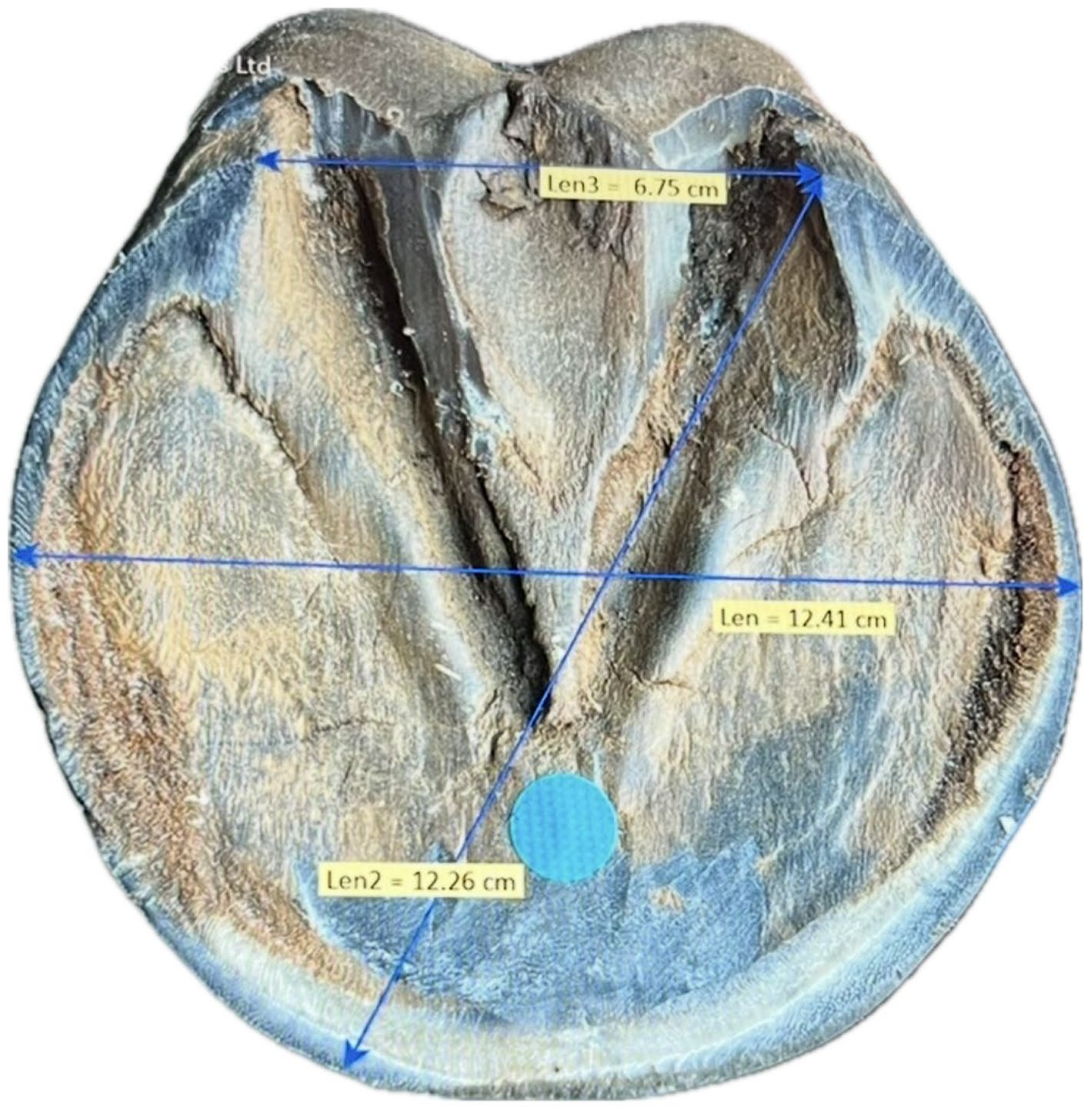
The solear metrics of hoof width, length & heel separation were measured in recognised software giving results in centimetres.

### Data analysis

Normality of the data was assessed using Shapiro–Wilk test before statistical analysis. Baseline comparisons between treatment and control groups were performed using independent-samples *t*-tests. Paired-samples *t*-tests were used to compare longitudinal measurements (initial versus endpoint) and pre-trim versus post-trim measurements within groups. To compare the magnitude of longitudinal change between treatment and control groups, horse-level change scores (endpoint minus initial values) were calculated for dorsal hoof wall angle (DHWA), heel angle (HA), and the difference between DHWA and HA, and compared using independent-samples *t*-tests. Pearson’s correlation analysis was used to assess the relationship between age and DHWA. Statistical significance was accepted at *p* < 0.05.

## Results

All variables were assessed for normality before analysis and met the assumptions required for parametric testing. Results are presented for baseline comparisons, longitudinal changes over the 90-day study period, immediate effects of trimming, and the relationship between age and dorsal hoof wall angle (DHWA).

### Age of horses

The mean age of the control group at the beginning of the study was 1,194 ± 268 days, and the mean age of the treatment group was 1,094 ± 230 days. The mean combined age of the treatment and control groups were 1,130 ± 245 days.

### Baseline comparison of groups

Independent-sample *t*-tests revealed no significant baseline differences between the treatment and control groups for DHWA (*p* = 0.579), HA (*p* = 0.368), DHWA-HA difference (*p* = 0.405), or hoof length (*p* = 0.062). The control group did exhibit greater hoof width at baseline than the treatment group (12.81 ± 0.81 cm vs. 12.11 ± 0.70 cm; *p* = 0.016).

### Effect of a 90-day unshod period

Comparison of horse-level change scores (Table 1) demonstrated a significantly greater increase in heel angle (HA) in unshod horses compared with shod controls (4.64 ± 4.64° versus 0.15 ± 5.10°; *p* = 0.015). No significant difference was identified between groups for changes in dorsal hoof wall angle (DHWA) (*p* = 0.129). The reduction in the discrepancy between DHWA and HA was greater in unshod horses (−4.56 ± 5.01°) than in shod controls (−1.00 ± 5.66°), although this did not reach statistical significance (*p* = 0.073).

**Table 1:** Comparison of changes over the study period.

Variable	Unshod mean change ± SD	Shod mean change ± SD	*p*
DHWA change (°)	0.09 ± 1.32	−0.85 ± 1.88	0.129
HA change (°)	4.64 ± 4.64	0.15 ± 5.10	0.015
DHWA–HA difference change (°)	−4.56 ± 5.01	−1.00 ± 5.66	0.073

### Treatment group

#### Mean pre-trim and post-trim

Descriptive statistics for hoof-angle measurements are presented in Table 2. In the treatment group, mean pre-trim HA increased from 29.97 ± 8.81° at the initial assessment to 34.62 ± 7.77° at the endpoint, while DHWA remained relatively stable (51.22 ± 3.28° to 51.31 ± 3.03°). The mean DHWA–HA difference decreased from 21.25 ± 6.90° to 16.69 ± 6.44°. These descriptive trends are supported by the change analysis presented in Table 1.

**Table 2:** Mean hoof angles were measured between the control and treatment groups at initial pre-trim, endpoint pre-trim, initial post-trim & endpoint post-trim.

Group	*n*=	Mean initial age	DHWA° mean	HA° mean	Difference mean DHWA - HA°
Control, initial pre-trim	26	1,194 ± 268	50.78	27.84	22.93
Treatment, initial pre-trim	46	1,094 ± 230	51.22	29.97	21.25
Control, endpoint pre-trim	26	1,194 ± 268	49.93	28.00	21.93
Treatment, endpoint pre-trim	46	1,094 ± 230	51.31	34.62	16.69
Combined, initial pre-trim	72	1,130 ± 245	51.06	29.2	21.86
Control, initial post-trim	26	1,194 ± 268	51.61	30.60	21.01
Treatment, initial post-trim	46	1,094 ± 230	51.67	32.2	19.50
Control, endpoint post-trim	26	1,194 ± 268	50.75	30.82	19.93
Treatment, endpoint post-trim	46	1,094 ± 230	51.32	36.11	15.21
Combined, initial post-trim	72	1,130 ± 245	51.65	31.6	20.04

Combined hoof angles indicate the control and treatment groups added together. N=: Number of hooves, DHWA: dorsal hoof wall angle, HA: heel angle.

### Hoof angles

Within the treatment group, HA increased significantly between the initial and endpoint assessments (*t* = −2.987, *p* = 0.005), whereas DHWA did not change significantly (*t* = −0.126, *p* = 0.900) (Table 3; Figure 5).

**Table 3:** Longitudinal changes in hoof morphology and hoof angles, and for immediate effects of trimming in treatment and control groups.

Comparison	*N*	*t*	df	*p*
Treatment: DHWA (Initial vs. Endpoint)	46	−0.126	45	0.900
Treatment: HA (Initial vs. Endpoint)	46	−2.987	45	0.005
Control: DHWA (Initial vs. Endpoint)	26	1.931	25	0.065
Control: HA (Initial vs. Endpoint)	26	−0.141	25	0.889
Treatment: DHWA-HA Difference (Initial vs. Endpoint)	46	3.984	45	0.001
Control: DHWA-HA Difference (Initial vs. Endpoint)	26	0.846	25	0.406
Treatment: DHWA-HA Difference LF	23	2.368	22	0.027
Treatment: DHWA-HA Difference RF	23	3.543	22	0.002
Control: DHWA-HA Difference LF	13	0.079	12	0.938
Control: DHWA-HA Difference RF	13	1.033	12	0.322
Treatment: Solear Sum (Pre- vs. Post-trim)	46	6.712	45	0.001
Treatment: Solear Width (Pre- vs. Post-trim)	46	6.303	45	0.001
Treatment: Solear Length (Pre- vs. Post-trim)	46	6.164	45	0.001
Treatment: Heel Width (Pre- vs. Post-trim)	46	2.980	45	0.002
Control: Solear Sum (Pre- vs. Post-trim)	26	2.943	25	0.003
Control: Solear Width (Pre- vs. Post-trim)	26	3.053	25	0.003
Control: Solear Length (Pre- vs. Post-trim)	26	3.129	25	0.002
Control: Heel Width (Pre- vs. Post-trim)	26	1.777	25	0.044
Combined: DHWA (Pre- vs. Post-trim)	72	−3.468	71	0.001
Combined: HA (Pre- vs. Post-trim)	72	−5.505	71	0.001
Combined: DHWA-HA Difference (Pre- vs. Post-trim)	72	4.173	71	0.001

N=: Number of hooves, DHWA: dorsal hoof wall angle, HA: heel angle, HW: heel width, LF: left fore and RF: right fore.

**Figure 5:**
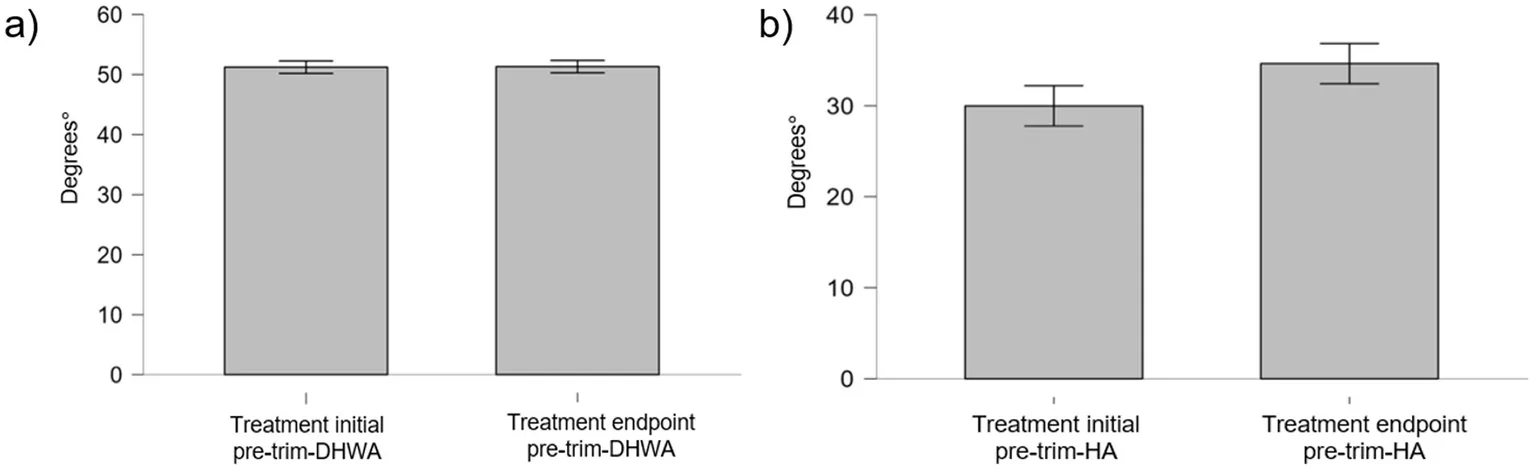
The treatment group showed a non-significant difference between **(a)** pre-trim DHWA initial to endpoint *t* = −0.126, *p* = 0.900 and **(b)** a very significant result between pre-trim HA initial to endpoint *t* = −2.987, *p* = 0.005.

The DHWA-HA difference decreased significantly between the initial and endpoint assessments (*t* = 3.984, *p* = 0.001), indicating a reduction in the disparity between dorsal hoof wall and heel angles (Table 3; Figure 6).

**Figure 6:**
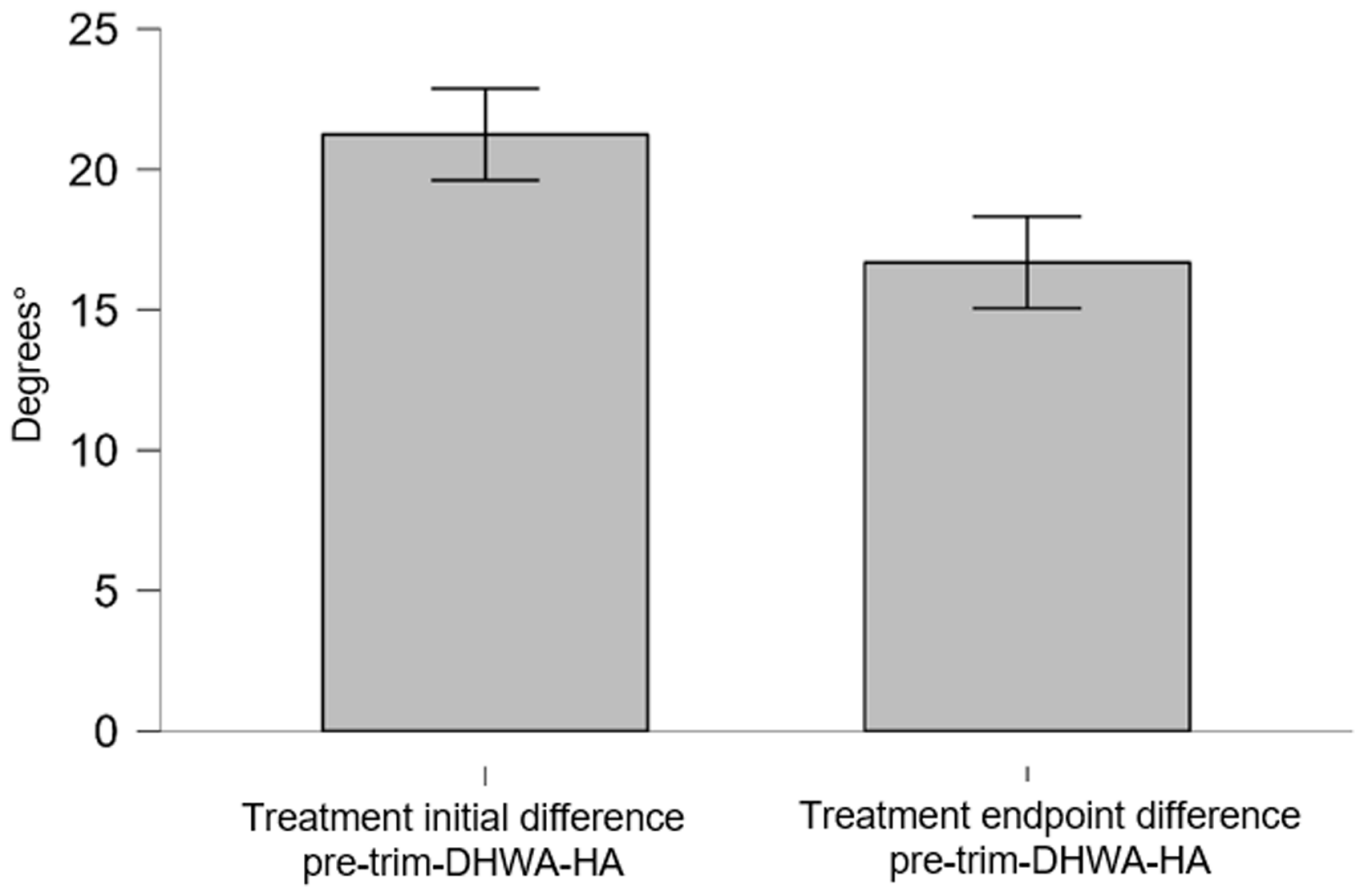
In the treatment group the difference between, initial pre-trim DHWA and initial pre-trim HA (DHWA-HA), compared to endpoint pre-trim DHWA and endpoint HA showed a highly significant reduction after a 90-day period unshod, *t* = 3.98, *p* = 0.001.

Analysis of individual forelimbs demonstrated significant reductions in DHWA-HA difference in both the LF (*t* = 2.368, *p* = 0.027) and RF (*t* = 3.543, *p* = 0.002) (Table 3; Figure 7).

**Figure 7:**
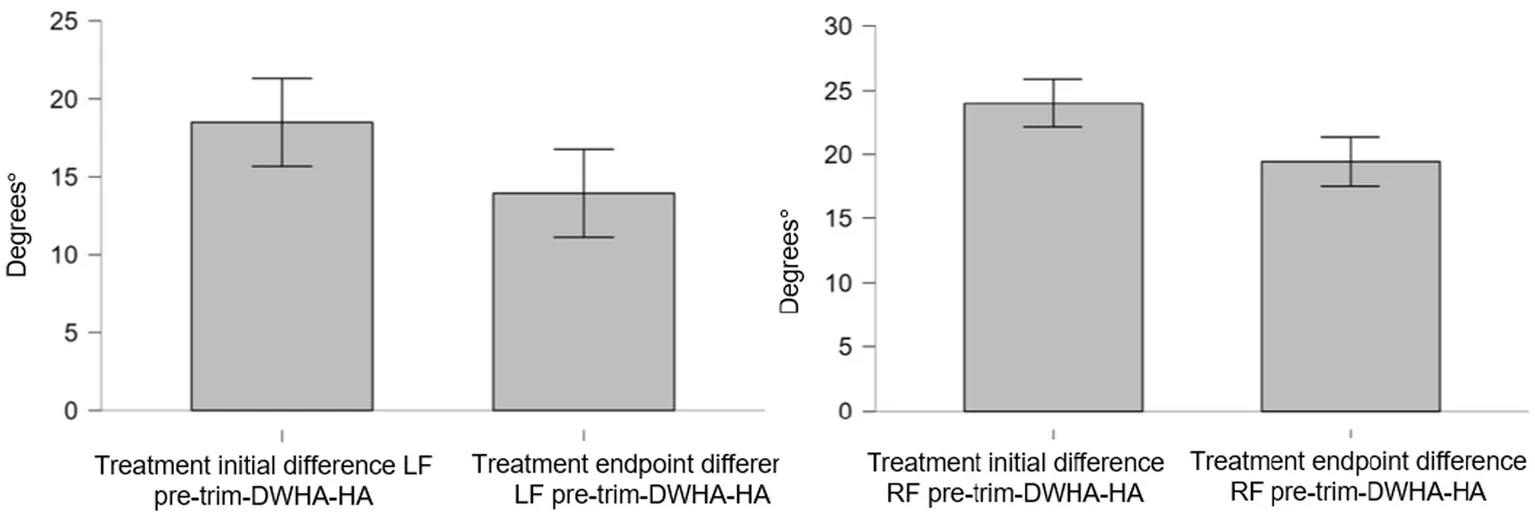
In the treatment group the difference between both left fore (LF) and right fore (RF) DHWA to HA reduced from the initial to endpoint, with the LF indicating a significant result *t* = 2.368, *p* = 0.027 and the RF a highly significant result, *t* = 3.543, *p* = 0.002.

### Control group

Descriptive statistics for hoof-angle measurements are presented in Table 2. In the control group, mean pre-trim DHWA changed from 50.78 ± 2.64° to 49.93 ± 3.29°, while mean HA changed from 27.84 ± 8.84° to 28.00 ± 8.87°. The mean DHWA-HA difference changed from 22.93 ± 8.00° to 21.93 ± 8.20.

### Hoof angles

Within the control group, no significant differences were detected for DHWA (*t* = 1.931, *p* = 0.065) or HA (*t* = −0.141, *p* = 0.889) between the initial and endpoint assessments (Table 3; Figure 8).

**Figure 8:**
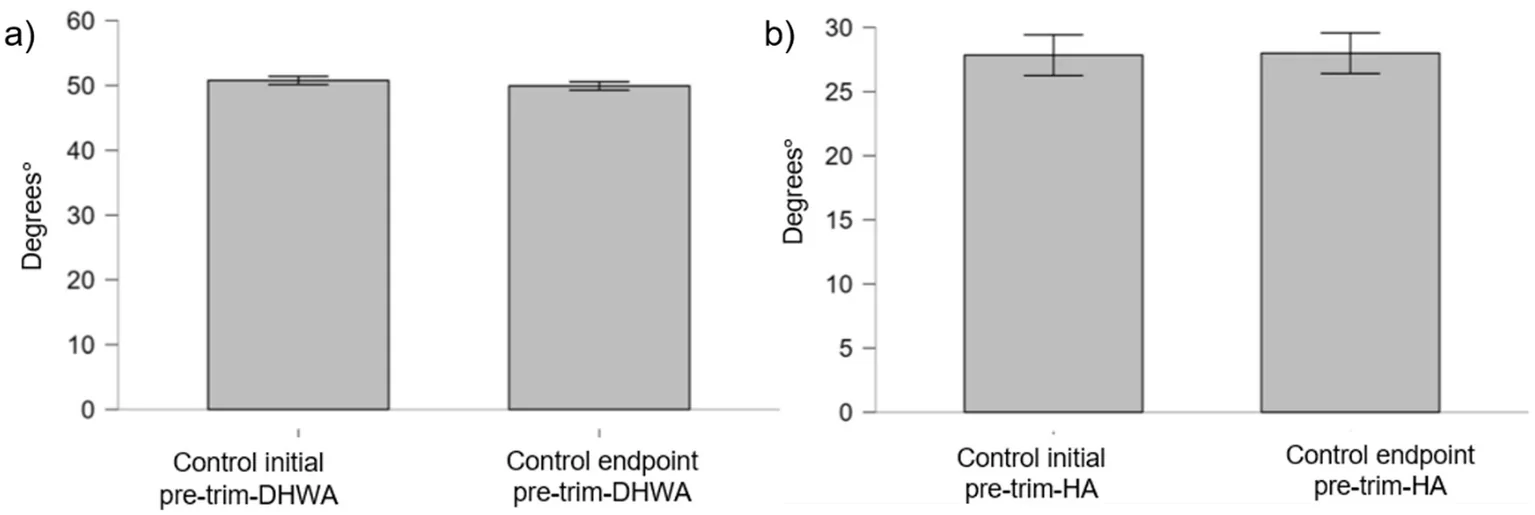
The control group showed a non-significant difference between **(a)** pre-trim DHWA initial to endpoint *t* = 1.931, *p* = 0.065 and **(b)** a non-significant result between pre-trim HA initial to endpoint *t* = −0.141, *p* = 0.889.

No significant change in DHWA-HA difference was observed between the initial and endpoint assessments (*t* = 0.846, *p* = 0.406) (Table 3; Figure 9).

**Figure 9:**
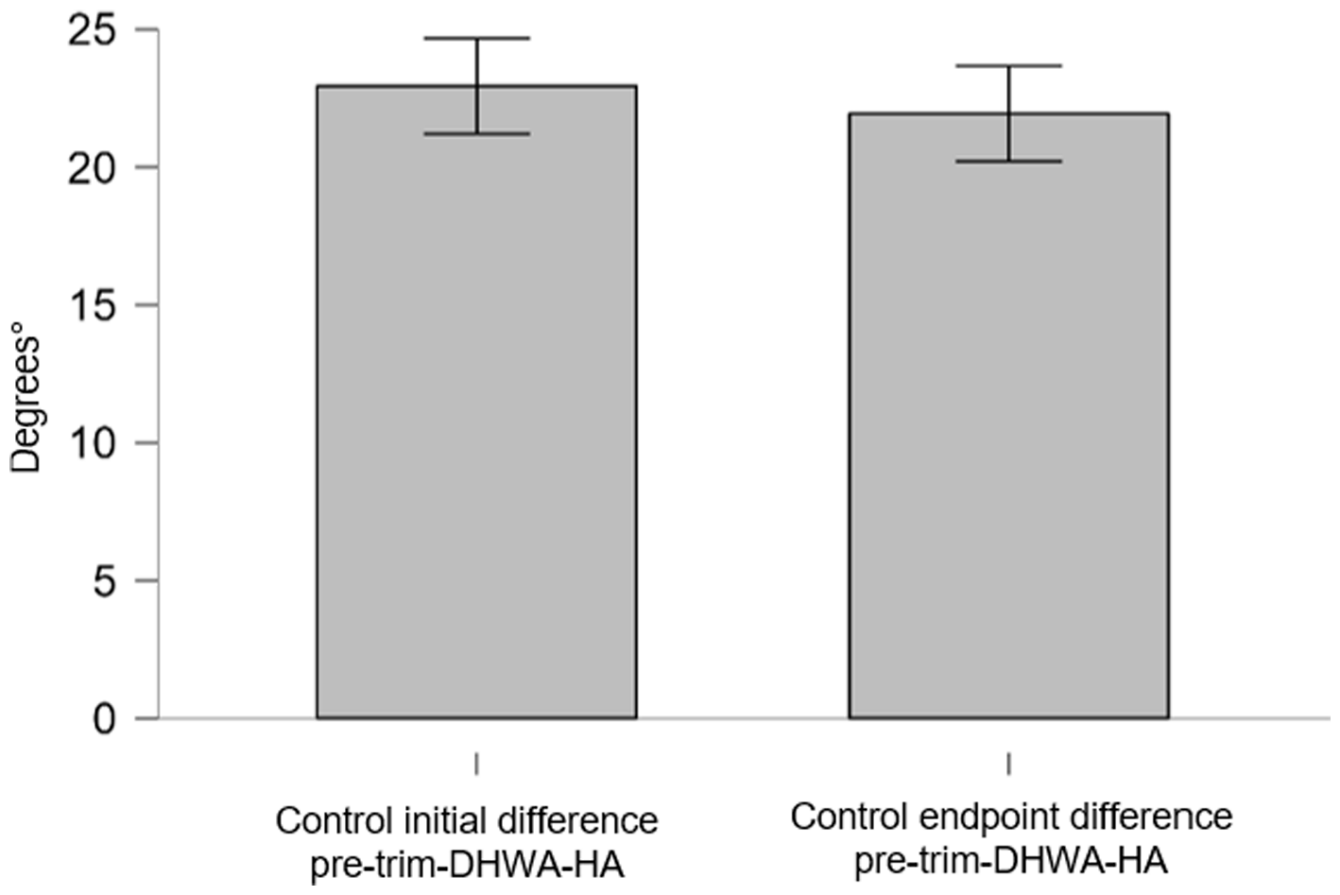
The control group result was non-significant between DHWA-HA difference, comparing initial to endpoint *t* = 0.846, *p* = 0.406.

### Solear morphometrics

Each solear image produced measurements of hoof width, hoof length, and heel width (HW). Descriptive statistics for pre-trim and post-trim morphometric measurements are presented in Table 4. In both the treatment and control groups, trimming reduced all measured dimensions, including width, length, and heel width.

**Table 4:** Mean initial solear pre-trim and post-trim morphometrics measured between the control and treatment groups.

Group	*N* (hooves)	Mean initial age	Solear width average in cm	Solear length average in cm	Solear heel width in cm
Control, initial pre-trim	26	1,194 ± 268	12.81	12.45	6.72
Treatment, initial pre-trim	46	1,094 ± 230	12.11	11.80	6.16
Control, initial post-trim	26	1,194 ± 268	12.43	12.10	6.52
Treatment, initial post-trim	46	1,094 ± 230	11.62	11.36	5.93

Significant differences between pre-trim and post-trim measurements for solear width, length, and heel width in both groups (Table 3). The treatment group showed significant reductions in solear sum dimensions (*t* = 6.712, *p* = 0.001), width (*t* = 6.303, *p* = 0.001), length (*t* = 6.164, *p* = 0.001) and heel width (*t* = 2.980, *p* = 0.002). Similar but smaller reductions were observed in the control group (Table 3).

### Combined control and treatment group angles

#### Mean pre-trim and post-trim

Descriptive statistics for the combined pre-trim and post-trim hoof-angle measurements are presented in Table 2. The combined dataset showed increases in both DHWA and HA after trimming, together with a reduction in the DHWA-HA difference.

#### Combined pre-trim to post trim

The combined dataset demonstrated an immediate effect of trimming on hoof angles. Following trimming, both DHWA and HA increased, while the difference between the two angles decreased (Table 2). Analysis of the combined treatment and control groups confirmed significant increases in DHWA (*t* = −3.468, *p* = 0.001) and HA (*t* = −5.505, *p* = 0.001), together with a significant reduction in the DHWA-HA difference (*t* = 4.173, *p* = 0.001) (Table 3; Figures 10a–c).

**Figure 10:**
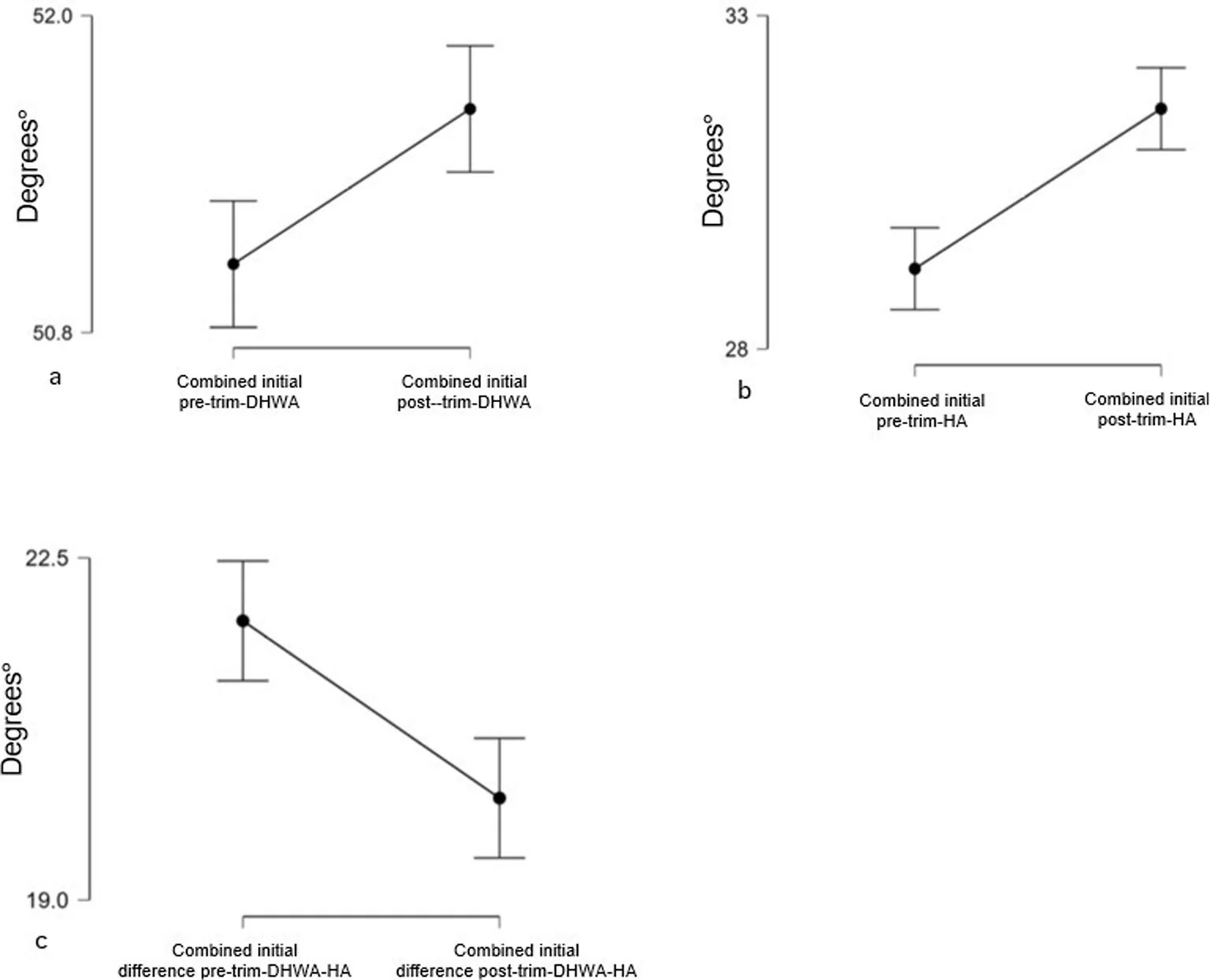
**(a–c)** Combined pre-trim versus post-trim hoof-angle comparisons. Trimming produced significant increases in DHWA and HA and significantly reduced the DHWA-HA difference (all *p* < 0.001).

### DHWA to age

A Pearson’s correlation analysis identified a moderate negative relationship between age in days and initial DHWA in both groups combined. As age increased, DHWA decreased (*r* = −0.412, *p* = 0.001).

## Discussion

This study demonstrated that significant morphological changes occurred during a 90-day period unshod whilst racehorses remained in active training. Unshod horses exhibited a significantly greater increase in heel angle than shod controls, while the disparity between DHWA and HA was also reduced. These findings support the first hypothesis that a 90-day period unshod may contribute to the restoration of hoof morphology associated with low heel conformation and dorso-palmar imbalance, traits previously associated with lameness and suspensory apparatus failure in Thoroughbred racehorses (1) thereby potentially helping to minimise hoof distortion and maintain balance (Figure 11).

**Figure 11:**
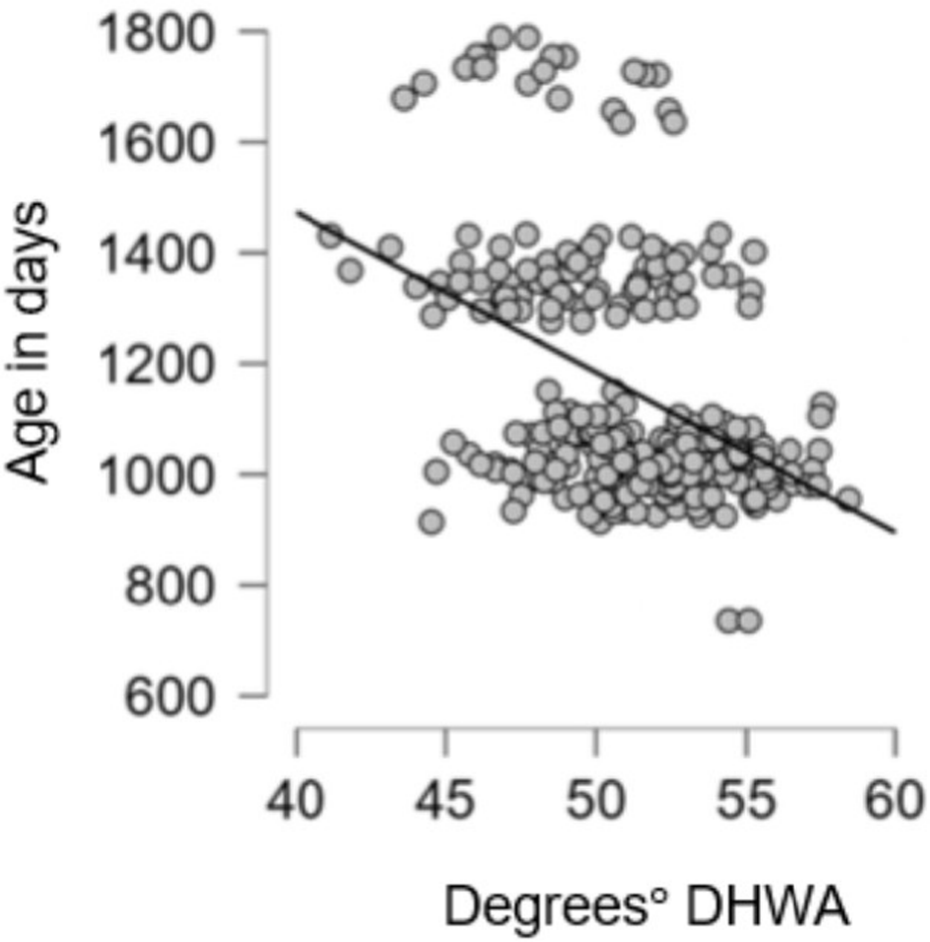
The downward sloping line indicates a negative correlation between age in days and DHWA, as age increased DHWA decreased, *r* = −0.412***, *p* = 0.001.

The increase in HA suggests that adaptation occurred predominantly within the palmar aspect of the hoof rather than through remodelling of the dorsal hoof wall. This interpretation is consistent with previous studies that reported improvements in heel support and hoof morphology following management strategies intended to increase frog and caudal hoof engagement during weight bearing (17). Increased contact between the frog, sole, and ground surface during the unshod period, whilst maintained through regular trimming, may have contributed to these changes and is consistent with hypotheses relating to stimulation of the digital cushion and associated structures (10, 18). Although the present study was not designed to directly assess these mechanisms, the findings suggest that restoration of hoof morphology may be more closely associated with improvement in heel angle than with alteration of the dorsal hoof wall angle.

The absence of significant morphological change in the control group suggests that routine management involving trimming and shoeing alone may be insufficient to support optimal hoof morphology during active training. One possible explanation is that the mechanical environment experienced by shod horses differs from that of unshod horses, particularly with respect to hoof-ground interaction and break-over dynamics (4). This interpretation is consistent with previous observations that prolonged shoeing may contribute to changes in heel conformation, digital cushion displacement, and caudal hoof function (9, 12), although further work is required to determine the specific mechanisms involved.

Leśniak (13) recommended a four-to-six-week shoeing cycle in working horses. In the present study, the control group remained shod on a 30-day cycle and did not demonstrate improvements in DHWA, HA, or the disparity between the two angles. This indicates that routine shoeing intervals are inadequate to reverse hoof-angle adaptations, as conventional open-heeled shoes elevate the hoof from the substrate, offering reduced support to the palmar hoof. Consequently, repetitive loading forces during intense race training could force the frog and digital cushion to deform between the heels of the shoe to contact the substrate, contributing to palmar hoof displacement and altered heel angles (9, 12). Although shorter hoof-care cycles may increase management costs, improvements in hoof morphology and balance could provide benefits for long-term soundness, performance and welfare. Alternatively, the improvements observed in the treatment group suggest that intermittent unshod periods may represent a beneficial management strategy for restoring hoof morphology in Thoroughbred racehorses, warranting further investigation under a wider range of training conditions.

While the present study was not designed to establish diagnostic thresholds for under-run heels in Thoroughbred racehorses, the results indicate that current accepted values may not fully reflect hoof morphology in actively training racehorses and may warrant further consideration. Mean DHWA-HA differences within this population were exceeded 21.86°, which is substantially greater than the 1–5° disparity commonly cited (1, 2, 14). These data indicate that current accepted diagnostic values do not reflect common dorso-palmar assessment of active training Thoroughbred racehorses, and support adopting a difference of ≥15° as a more realistic benchmark for identifying under-run heels amongst Thoroughbred racing populations. Further investigation using larger populations would be required before revised thresholds could be recommended; however, these findings highlight the potential value of reassessing how heel conformation is evaluated within racing populations. This could lead to adjustments that may result in significant changes to hoof-care approaches, digital support strategies and training schedules in an attempt to address mechanical breakdown and counterpart early retirement associated with these disparities (1, 3).

A greater ratio of adipose tissue relative to fibrocartilage has been reported within the digital cushion of young horses (10). As the present study focused on horses aged two to four years from the outset, developmental changes in the digital cushion may have influenced hoof morphology during the study period. This lower structural firmness may partly explain why low heel conformation and under-run heels are commonly observed in young shod racehorses. It is therefore possible that shoeing practices introduced at an early age influence subsequent hoof morphology. Further investigation should focus on determining the extent of this effect and whether alternative farriery practices, such as incorporating enhanced palmar support, might mitigate these developmental effects.

The analysis confirmed that the trimming protocol produced immediate changes in hoof angle, size and shape across both the treatment and control groups. Whilst HA increased immediately following trimming in both groups, only the unshod horses demonstrated a sustained improvement over the 90-day study period. This suggests that morphological deterioration occurring between trimming intervals may have been greater in the shod horses, thereby reducing the benefits gained from trimming alone.

The moderate negative correlation identified between age and DHWA suggests that DHWA continues to decrease between the ages of two and five years. This extends previous observations reported by Anderson et al. (11) and Curtis (5), which primarily focused on younger horses. These findings indicate that age should be considered when interpreting hoof-angle measurements in Thoroughbreds, as values considered typical in younger horses may not be directly applicable to older horses. Establishing age-specific reference values for monitoring hoof conformation would therefore improve early identification of horses at risk of adverse morphological adaptations during active training.

The observed adaptations are consistent with previous studies suggesting that increased hoof-ground interaction, frog engagement and palmar hoof loading may influence hoof morphology (3, 9, 10, 17). While the present study did not directly investigate the underlying mechanisms, the improvements in the treatment group indicate that increased palmar hoof substrate contact provides a necessary mechanical stimulus to counter heel collapse.

## Conclusion

This study demonstrated that a 90-day period of being unshod was associated with improved heel angle and a reduction in the DHWA-HA discrepancy, supporting the first hypothesis, that morphological and hoof angle changes can be restored during a period of being unshod. The significantly greater increase in HA observed in the treatment group compared with shod controls further strengthens the interpretation that these changes were associated with increased palmar hoof contact with the substrate, combined with regular trimming. Consequently, integrating strategic barefoot periods into training programs could offer a non-invasive method to reverse DHWA-HA disparity, a metric often associated with musculoskeletal injuries, early retirement, and financial expenses incurred due to lameness.

The data from both groups support the second hypothesis, providing compelling evidence that trimming immediately affects hoof angle and that the difference between DHWA and HA can be improved with regular farrier intervention. Although improvements were seen from pre-trim to post-trim in both groups, the shod controls showed no hoof angle improvements over the course of the 90-day study. This suggests that once a Thoroughbred racehorse is shod, heel angle deteriorates over the course of a 30-day hoof care cycle, adding to evidence that palmar hoof interaction with the substrate is important to sustain morphological responses and supports shortening shod racehorse shoeing cycles.

Furthermore, the observation that the DHWA continues to descend until at least five years old, as a natural consequence of ageing, builds on our existing knowledge. This could provide information leading to age-defined hoof angle grading systems for racehorses. In addition, the data point to a new benchmark for analysing under-run heels of ≥15° in Thoroughbred racehorses, highlighting a need for further investigation regarding normal hoof conformation in racing populations. Crucially these findings advocate prioritising palmar hoof loading and substrate interaction, rather than common practices involving manipulating the dorsal hoof wall to achieve improved hoof morphology. Importantly, barefoot management should not be regarded as universally appropriate. Practitioners should monitor individual horses closely and, where hoof wear exceeds growth or compromises soundness appropriate hoof protection or shoeing should be reinstated to safeguard equine welfare.

## Data Availability

The original contributions presented in the study are included in the article/Supplementary material, further inquiries can be directed to the corresponding author/s.
