# Supplementary material for: Changes in hoof morphology in racing Thoroughbreds during a period of ninety days unshod
Source: Front Vet Sci. 2026 Oct 1;13:1863433. doi: 10.3389/fvets.2026.1863433 (PMC13668592; doi:10.3389/fvets.2026.1863433)
Supplement: Supplementary file 1 [file Supplementary_file_1.pdf]

Appendix 1

Shapiro-Wilk test table of results show all p-values greater than 0.05 proving all data was normally distributed apart from control pre-trim solear length.

Initial group	Shapiro-Wilk	p=value of Shapiro-Wilk
Treatment pre-trim DHWA	0.976	0.438
Treatment pre-trim HA	0.976	0.451
Treatment post-trim DHWA	0.977	0.475
Treatment post-trim HA	0.967	0.209
Treatment pre-trim solear width	0.985	0.823
Treatment pre-trim solear length	0.976	0.452
Treatment pre-trim solear HW	0.987	0.891
Treatment post-trim solar width	0.960	0.116
Treatment post-trim solar length	0.979	0.567
Treatment post-trim solear HW	0.969	0.253
Control pre-trim DHWA	0.926	0.062
Control pre-trim HA	0.972	0.670
Control post-trim DHWA	0.982	0.905
Control post-trim HA	0.969	0.586
Control pre-trim solear width	0.937	0.115
Control pre-trim solear length	0.868	0.003
Control pre-trim solear HW	0.953	0.269
Control post-trim solear width	0.932	0.087
Control post-trim solear length	0.939	0.127
Control post-trim solear HW	0.884	0.007
Combined pre-trim DHWA	0.974	0.137
Combined pre-trim HA	0.991	0.889
Combined pre-trim DHWA to HA difference	0.986	0.621
Combined post-trim DHWA	0.982	0.413
Combined post-trim HA	0.985	0.555

Combined post-trim DHWA to HA difference	0.987	0.651

Appendix 2

Manufacturers addresses

1. iPhone 14, Apple Inc. One Apple Park Way, Cupertino, CA 95014 USA

2. Metron Mind, 6720 Line Road, Pass Robles, CA 93446, USA

3. Apple Inc. One Apple Park Way, Cupertino, CA 95014 USA -Apple Macbook

4. Calibration stickers - Tindalls 50-52 High Street,
Newmarket Suffolk, CB8 8LE

5. ASUSTeK Computer Inc. No.150, LI-Te Rd, Peitou, Taipei 11259, Taiwan

6. Excel - Microsoft, One Microsoft Way, Redmond, Washington 90526399

7. Jasp statistical software - Nieuwe Achtergracht
129B, Amsterdam, The Netherlands

Acknowledgments

R Beaver, R Nelson, J Burnett Wells, for horse handling management and assistance.
